# Evaluation of Dutasteride-Loaded Liposomes and Transfersomes for Follicular-Targeting for Androgenic Alopecia Topical Treatment

**DOI:** 10.3390/pharmaceutics16121524

**Published:** 2024-11-27

**Authors:** Jayanaraian F. M. Andrade, Breno N. Matos, Rafael V. Rocho, Geisa N. Barbalho, Marcilio Cunha-Filho, Guilherme M. Gelfuso, Taís Gratieri

**Affiliations:** Laboratory of Food, Drugs, and Cosmetics (LTMAC), University of Brasilia (UnB), Brasília 70910-900, DF, Brazil; mjayanaraian@gmail.com (J.F.M.A.); brenomatos15@hotmail.com (B.N.M.); rafael.rocho@aluno.unb.br (R.V.R.); geisabarbalho@gmail.com (G.N.B.); marciliofarm@hotmail.com (M.C.-F.); gmgelfuso@unb.br (G.M.G.)

**Keywords:** androgenic alopecia, dutasteride, follicular targeting, liposome, transfersome

## Abstract

**Background/Objectives:** Although androgenic alopecia is the most prevalent among non-cicatricial alopecia, it still lacks an effective and safe treatment. Dutasteride (DUT) shows promising results in hair regrowth; however, oral DUT intake causes serious sexual adverse events. Hence, we produced liposomes with different bilayer structures and evaluated the capability of such systems in increasing DUT accumulation in the hair follicles. **Methods:** In vitro skin penetration tests were performed with porcine ear skin, and the follicular targeting factor (Tf) was calculated as the ratio between DUT amount in HFs and DUT recovered from the sum of all skin layers. **Results:** While the stiffer DUT-loaded liposome was not able to target the hair follicles in 12 h (Tf = 0.15), a DUT-loaded liposome with an edge activator in its composition, i.e., transfersomes, promoted better control over DUT release and a higher Tf (0.32) (*p* < 0.005). **Conclusions:** Transfersomes present higher affinity with DUT providing a better controlled release; hence, they are a better option for DUT follicle targeting compared to liposomes. Further formulation optimizations are needed aiming to prolong such targeting effect.

## 1. Introduction

Androgenetic alopecia is the most common form of hair loss, affecting 80% of men and 50% of women under 70 years of age [1,2]. Although it is not a life-threatening disease, it is crucial to consider the psychological impact of this condition on patients. Many patients face body dysmorphic disorder and experience a significant reduction in self-esteem, potentially developing illnesses such as anxiety and depression [3,4]. Although alopecia is a long-known condition, the treatments available are limited.

Current treatment of androgenetic alopecia comprises mainly oral finasteride, a 5-alpha reductase inhibitor, and topical minoxidil, which promotes hair growth, prolonging the anagen phase, but does not stop the balding process [5]. Moreover, both present meaningful limitations. While systemic absorption of finasteride can cause sexual adverse effects, such as erectile and ejaculatory dysfunction, sexual impotence, and gynecomastia [6], minoxidil has low water solubility (2 mg/mL), which comes with a series of technical difficulties for use. Due to its characteristics, minoxidil is delivered in solutions with high ethanol and/or propylene glycol content, substances that are extremely irritating to the skin after repeated applications [7,8,9]. In addition, these volatile vehicles tend to evaporate, resulting in minoxidil crystallizing on the scalp, preventing its penetration [10,11].

Dutasteride (DUT) has been explored as a more effective option for alopecia treatment [12]. DUT belongs to the same class as finasteride, but because it is more potent, it can be administered in smaller and less frequent doses, contributing to treatment adherence. Indeed, DUT resulted in superior efficacy in hair regrowth compared to finasteride in different clinical trials [13,14,15,16]. However, DUT also has a great potential to cause the same adverse effects as finasteride, with the aggravating factor of a longer half-life of approximately 5 weeks [17]. For this reason, DUT is only FDA-approved to treat benign prostatic hyperplasia until this moment. Thus, developing a topical DUT non-irritant formulation that drives the drug to the hair follicles (HFs), reducing systemic absorption and consequently minimizing adverse effects, rises as a promising androgenetic alopecia treatment option. However, DUT’s physicochemical characteristics make the development of such a drug delivery system challenging.

DUT is a very lipophilic drug (log P = 6.8), which prevents it from being incorporated into conventional aqueous formulations. On the other hand, DUT incorporation into oily vehicles would result in a product with a heavy and undesirable appearance to the hair [18]. Therefore, DUT requires a technological alternative to be delivered effectively. In this context, liposomes are among the most versatile drug delivery systems for encapsulating lipophilic drugs due to their amphiphilic character.

Structure-wise, liposomes are lipid bilayer vesicles with an aqueous core dispersed in an aqueous medium so that they can encapsulate both lipophilic and hydrophilic molecules [19,20]. Transfersomes (TFs) are a type of liposome with a more flexible vesicle structure, as they contain an edge activator in their composition. Both liposomes and TFs are biocompatible, biodegradable, and safe for topical application [21,22,23]. These structures have been described to sustain drug release and interact with the lipids of the stratum corneum and the sebum of HFs [24], resulting in a film formation with an occlusive effect, providing a prolonged contact time between the formulation and the target site, increasing drug penetration [25,26,27]. These advantages are probably why, even though the FDA still does not approve DUT for topical use in alopecia treatment, it has a considerable off-label use [13,15,28] with formulations sold in compounding pharmacies under prescription.

Previous work demonstrated the superiority of a liposome formulation in promoting a higher DUT skin permeation than a hydroalcoholic DUT solution and conventional gels [29]. However, the mentioned work used mouse skin as a model, which is more permeable than human and porcine skin, and did verify DUT accumulation in HFs. In fact, for the effective topical treatment of alopecia, the formulation must target the HFs to limit systemic drug exposure.

Hence, this work aimed to develop DUT-loaded liposomes with different bilayer structures, including a conventional liposome obtained with phosphatidylcholine (LP PC), a stiffer liposome with cholesterol in its composition (LP Chol), and a flexible liposome, also denominated TFs, to evaluate the capability of such systems in targeting HFs, increasing DUT accumulation in these structures. An oily DUT solution was used as a control.

## 2. Material and Methods

### 2.1. Material

DUT was purchased from Henan Tianfu Chemical Co. (Zhengzhou, China). Phosphatidylcholine (PC) from soybean was purchased from Lipoid (S 100 PC ≥ 94.0%, Ludwigshafen, RP, Germany). Methanol, Chol, Tween^®^ 80, and sodium acetate were purchased from Sigma-Aldrich (St. Louis, MO, USA). Sodium dodecyl sulfate (SDS), chloroform, and acetic acid were purchased from Dinâmica (São Paulo, Brazil). Acetonitrile was purchased from J. T. Baker (Phillipsburg, NJ, USA). Glucam N was purchased from Biovital (São Carlos, Brazil). Scotch n^o^. 845 Book Tape was purchased from 3 M (St. Paul, MN, USA). Cyanoacrylate glue was purchased from Henkel Loctite (Dublin, Ireland). Water was purified using a Milli-Q system (Millipore, MA, USA) with a 0.22 µm pore end filter. All other chemicals and reagents were of analytical grade.

The porcine ears were obtained from a local slaughterhouse (Suino Bom Alimentos Ltd., Brasilia, Brazil) shortly after animal sacrifice. In all the experiments, full-thickness porcine skin was used (thickness of approximately 2.0 mm). This means the subcutaneous fat was removed, leaving the entire dermis intact. In this way, all the epidermal and dermal skin layers were intact, ensuring the hair follicles were not harmed in any way. Skin samples removed from the ears and cleansed were stored at −20 °C for a maximum of 1 month.

### 2.2. Liposome Formation

Liposomes were obtained by the lipid film hydration technique [24]. Briefly, stock solutions of DUT (28 mM), PC (200 mM), and Tween^®^ 80 (200 mM) were prepared in methanol, while a Chol (10 mM) solution was prepared in chloroform. Liposome composition is listed in Table 1.

The lipidic films were formed by roto evaporation (IKA Lab Dancer, IKA, Staufen, Germany) at 60 °C for 30 min. Films were then hydrated for 1 h with 10 mL acetate buffer (pH 5.4). After hydration, the formulation was homogenized for 30 min at 14,500 rpm in Turrax^®^ RW 20 (IKA Lab Dancer, Staufen, Germany).

Since DUT is not soluble in the acetate buffer medium (water solubility: 0.908 µg/mL), the unloaded drug formed large crystals. Liposome dispersions were filtered with polytetrafluoroethylene (PTFE) hydrophilic filters with 0.45 µm pore size to remove these crystals from samples. The filtered liposomes were used in all experiments.

### 2.3. Characterization of DUT-Loaded Liposomes

#### 2.3.1. Liposome Size and Zeta Potential

Hydrodynamic diameter and polydispersity index (PdI) were determined by dynamic light scattering and zeta potential by electrophoretic mobility (Zetasizer Nanoseries, Malvern Instruments, Worcestershire, UK) using the samples diluted in purified water (1:100 *v*/*v*) at 25 °C. All measurements were performed in triplicate.

#### 2.3.2. Encapsulation Efficiency

For total DUT quantification in formulations and control, 100 µL of samples were diluted in methanol (1:10 *v*/*v*) and vortexed for 60 s. To determine the encapsulation efficiency (EE%), aliquots of liposomes were taken before and after liposome filtration. EE% was calculated as follows (Equation (1)):

(1) $$EE\%=\frac{{Total\;DUT\;amount}_{filtered}}{{Total\;DUT\;amount}_{\;unfiltered}}\times 100$$

#### 2.3.3. Morphological Analyses

*Transmission electron microscopy:* Morphological analyses were performed using a transmission electron microscope (TEM; JEM 1011 Transmission Electron Microscope, JEOL, Tokyo, Japan—100 kV). Images were captured with a GATAN BioScan camera (model 820, GATAN, Warrendale, PA, USA) using the Digital Micrograph 3.6.5 software (GATAN, PA, USA). Samples were prepared as previously reported. Briefly, diluted aliquots of filtered liposomes were deposited on a Formvar-coated copper grid (Electron Microscopy Sciences, Hatfield, PA, USA) and air-dried for 10 min. The excess of the formulations was absolved by filter paper. After that, a 3% uranyl acetate solution (*w*/*v*) was added and air-dried for 10 min, and the excess was also cleaned with filter paper.

*Polarized light microscopy:* The formulations were also analyzed by polarized light microscopy (PLM, Axio Imager Z2m, Carl Zeiss, Oberkochen, Germany) equipped with an Axiocam 305 camera to verify the presence of DUT nanocrystals. Samples were prepared by putting one drop of each formulation on a glass slide and covering it with a coverslip.

### 2.4. Drug Release

In vitro drug release was determined using cellulose membranes mounted in a Hanson diffusion cell assembly (Teledyne Hanson Research Inc., San Francisco, CA, USA) (diffusional area = 1.77 cm^2^). All tested formulations and the control contained approximately 0.30 mg/mL of DUT and were performed in quintuplicate. The donor compartment was filled with 500 µL of each formulation, while the receptor compartment contained 15 mL of 59.5% water/0.5% Tween 80/40% ethylene glycol solution to ensure sink conditions. The system was kept under magnetic stirring (300 rpm). Samples (1 mL) were withdrawn from the acceptor solution every hour for 12 h and then at 24, 36, and 48 h for DUT quantification, while the same volume of fresh solution was replaced in the acceptor compartment. A DUT oil solution was used as a control.

### 2.5. In Vitro Skin Penetration Tests

In vitro penetration tests were conducted with porcine ear skin mounted in a Hanson diffusion cell assembly (diffusional area = 1.77 cm^2^). The receptor compartment was filled with 15 mL of 0.5% SDS aqueous solution, and 500 µL of each test sample was placed in the donor compartment in contact with the stratum corneum for 12 or 24 h. At the end of each treatment, DUT was extracted from the skin layers using the differential stripping technique [24]. Briefly, at the end of the experiments, each skin sample was placed on a flat surface with the stratum corneum facing upwards. The stratum corneum was removed by the application of 15 adhesive tapes. Following, hair follicles were removed by placing a drop of cyanoacrylate glue in the same skin area and covering it with another adhesive tape with light pressure. After glue polymerization (±2 min), the tape was removed. For complete hair follicle removal, this process was performed twice. Finally, the remaining skin was cut into pieces with scissors. Drug extraction was carried out by adding 2.5 mL of methanol under rotation in a multi-rotator (model Multi Bio RS-24, BioSan, Riga, Latvia) for 24 h.

The follicular targeting factor was assessed by the following Equation (2):

(2) $$T_{factor}=\frac{{DUT\;}_{HF}}{\sum DUT\;penetrated}$$

where “*DUT_HF_*” is the DUT amount recovered from the HFs and “∑*DUT_penetrated_*” is the sum of the DUT amount recovered from all skin layers, i.e., stratum corneum, HFs, and viable skin.

### 2.6. Analytical Method

DUT quantification was performed by HPLC (model LC-20 CE, Shimadzu, Kyoto, Japan) with UV detection (SPD-M20A) at 242 nm. A reverse-phase Luna C8(2) column (150 × 4.6 mm; 5.0 μm, Phenomenex^®^) was used. The mobile phase consisted of Mili-Q water/acetonitrile (47:53 *v*/*v*) in the isocratic mode under a 1 mL/min flow rate at 40 °C. The injection volume of samples was 20 μL. This method was validated following International Conference on Harmonization (ICH) guidelines and was selective and linear in the concentration range of 0.25–120.0 µg/mL (r = 0.9999; y = 22,858x + 3635.2). The intra- and inter-day precision and accuracy of the method showed a coefficient variation (CV%) and a relative error (E%) < 5%. The limit of detection (LOD) and limit of quantification (LOQ) were 0.14 and 0.41 µg/mL, respectively. DUT recovery from the stratum corneum, HFs, and viable skin (viable epidermis and dermis) were previously determined with values higher than 97%.

### 2.7. Statistical Analysis

The statistical significance of the data was evaluated by analysis of the variance (ANOVA) with Tukey’s post hoc test. The significance level was fixed at 0.05. All data were expressed as mean ± standard deviation.

## 3. Results and Discussion

### 3.1. Liposome Characterization

Here, four formulations were compared: an oily solution used as a control and three liposomes with different vesicle structures. The first liposomal formulation (LP PC) is a simple liposome formed by soybean phosphatidylcholine (PC), the main base of liposomes [30,31]. The second liposome, in addition to PC, contains cholesterol (Chol), which is commonly employed to increase liposomes’ stability and biocompatibility [32,33]. It also promotes a condensing effect on the phospholipid bilayer, creating more rigid vesicles [34]. The third liposome, in addition to PC, contains an edge activator forming a special kind of liposome, also known as TF. The edge activator, a surfactant, makes smaller and ultra-deformable vesicles up to 8 times more elastic than a conventional liposome [35,36].

**Figure 1:**
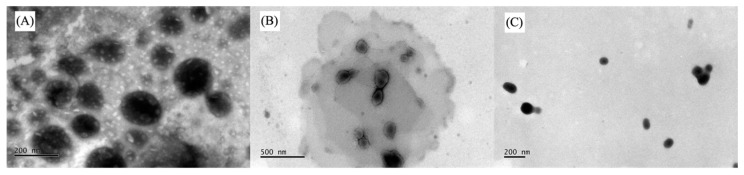
TEM images of the DUT-loaded liposomes: (**A**) LP PC, 10 k; (**B**) LP PC Chol, 25 k; and (**C**) TF, 12 k.

As expected, the morphological analysis by TEM showed that TF had a condensed core and formed smaller vesicles than the other liposomes (Figure 1C), even smaller than DLS size measurement (Table 2), which some aggregation in the sample can explain. LP PC also formed spherical vesicles with a condensed core but bigger than the other liposomes (Figure 1A). LP PC Chol presented a more irregular shape (Figure 1B), which can be attributed to Chol’s concentration, directly impacting liposome phospholipidic membranes. This behavior has been previously demonstrated in the literature, in which increasing Chol’s concentration caused irregular-shaped liposomes to become spheric [37,38,39].

**Figure 2:**
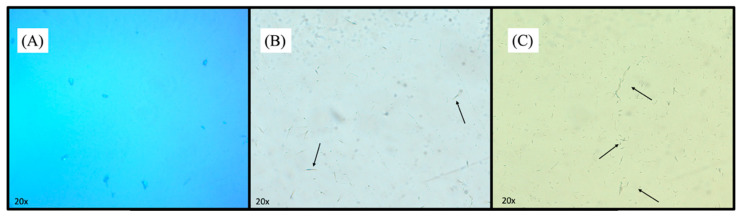
Polarized light microscopy of DUT-loaded liposomes. (**A**) Unfiltered empty liposome; (**B**) Unfiltered DUT-loaded liposome—TF; and(**C**) Filtered DUT-loaded liposomes—TF. Magnification: 20×.

As mentioned, DUT is a very lipophilic molecule (water solubility: 0.908 µg/mL, log P = 6.8). Hence, in such colloidal dispersions, the drug is expected to be encapsulated, specifically between the lipidic bilayer. The drug amount that exceeds the loading capacity of the formulation would not be soluble and precipitate. However, no precipitates were formed in any of the formulations. In this way, we hypothesized that DUT could have formed insoluble colloidal crystals that could be either in the internal aqueous core or the external aqueous medium. To confirm or reject this hypothesis, the formulations were visualized under a polarized light microscope. Unfiltered empty liposomes were also observed as controls (Figure 2A). Indeed, nothing is observed in the formulations without the drug, but in unfiltered samples containing DUT, many narrow filaments were identified as crystals (Figure 2B). These filaments are characterized by a shift in the reflected color light when the microscope glass is moved. The filtration process through a 0.45 µm pore syringe filter removed a portion of crystals and mainly decreased the crystals’ size, but some smaller crystals still passed through the 0.45 µm pore syringe filter, as seen in Figure 2B,C. The size measurements, PdI reductions, and EE% after homogenization and filtration further confirm crystals’ removal and downsizing (Table 3).

Because the presence of insoluble DUT crystals could interfere with the analysis of the influence of the liposome vesicle characteristics for the HF targeting effect, filtered samples were used for the subsequent tests. It is essential to highlight that all the samples were produced to achieve a similar DUT final concentration (approximately 0.30 mg/mL). The encapsulation efficiency (EE%) was considered the ratio between unfiltered and filtered samples reaching such DUT final concentrations (Table 2).

### 3.2. In Vitro Drug Release

**Figure 3:**
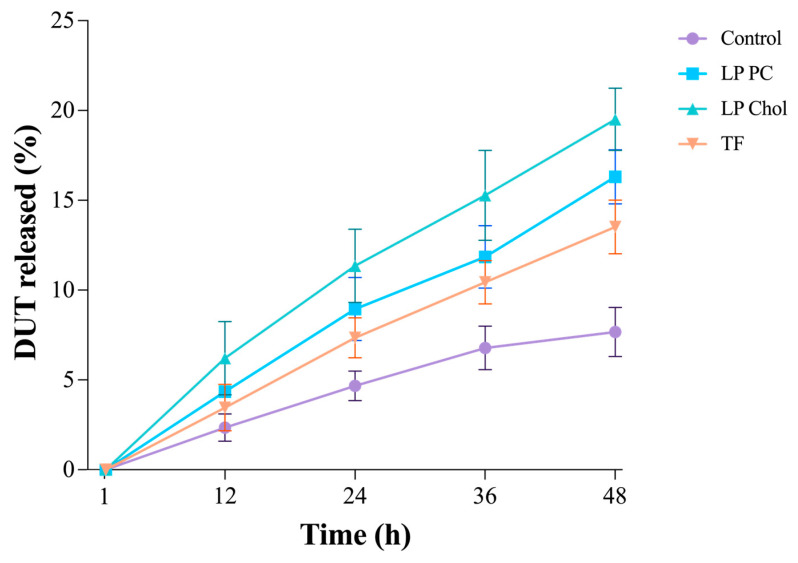
DUT release from the liposomes LP PC, LP Chol, and TF, and control (DUT oily solution) over 48 h. Data expressed as mean ± SD (n = 5).

Figure 3 shows the DUT release percentage from the liposomes and control—a DUT oily solution. The oily solution would not be a feasible formulation for topical delivery because of the greasy aspect it would leave the scalp. However, it was chosen as the control for being a formulation that would completely dissolve DUT, hence with a higher affinity for it.

All liposomes showed prolonged release profiles, but TF was the formulation that mostly approached the oily solution. The edge activator presence seemed to enhance DUT solubilization, increasing DUT’s release time. TF presented only 13.5 ± 1.5% drug release, while LP PC released significantly more drug (*p* < 0.0001) in the same period, 16.3 ± 1.5%. After 48 h, LP PC Chol had the highest DUT released amount of 19.5 ± 1.7% (*p* < 0.0001). In fact, Chol (log P = 7.0) is as lipophilic as DUT (log P = 6.8); therefore, both molecules naturally compete to interact with the liposomes’ lipids, causing DUT to be released from this liposome faster than the others.

### 3.3. In Vitro Skin Penetration Experiment and Follicular Targeting Effect

**Figure 4:**
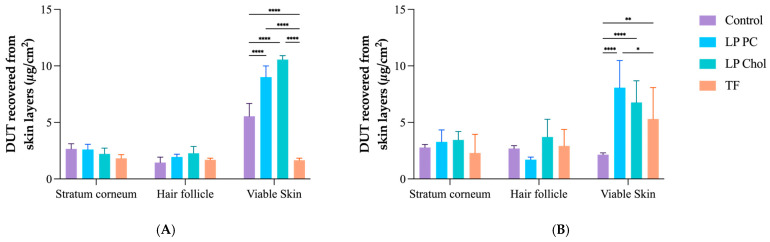
DUT amount recovered from the stratum corneum, hair follicles, and viable skin after 12 h (**A**) and 24 h (**B**) of in vitro skin penetration experiments with the liposomes LP PC, LP Chol, TF, and control. Data expressed as mean ± SD (n = 6). **** *p* < 0.0001, ** *p* < 0.007, and * *p* < 0.02.

**Figure 5:**
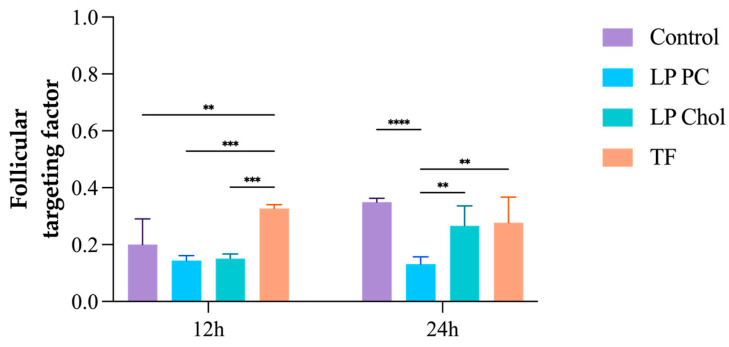
Follicular targeting factor of DUT from the liposomes LP PC, LP Chol, TF, and control after 12 and 24 h of skin application. **** *p* < 0.0001, *** *p* < 0.0008, and ** *p* < 0.005.

Skin penetration tests were performed for 12 and 24 h using skin obtained from porcine ears. In 12 h, LP PC Chol promoted more significant DUT accumulation in the HFs (2.28 ± 0.60 µg/cm^2^), 1.2 and 1.3 times higher than LP PC and TF, respectively. Although TF had lower DUT accumulation amounts in all skin layers (Figure 4) in this period, it had a more significant effect in targeting HFs, i.e., the ratio between DUT amount in HFs and DUT recovered from the sum of all skin layers was 0.32 (*p* < 0.005), compared to LP PC and LP PC Chol, which had 0.14 and 0.15 targeting factors, respectively (Figure 5). The follicular targeting potential of TF in 12 h is similar to other DUT-loaded delivery systems found in the literature. In the same period of skin treatment, lipid-core nanocapsules with and without chitosan coating had a targeting factor of approximately 0.28 [18].

The results presented here show a slight superiority of elastic vesicles in follicular targeting in a shorter period. However, drug distribution among the skin layers is equilibrated in 24 h. Indeed, the TF targeting factor decreases to 0.27 in a prolonged time. In fact, TF targets the HF following the administration. Still, with time, the drug is either released from these structures and continues to diffuse from the HF to other skin layers, or the entire TF structure continues to penetrate the skin from the HF. Even though the latter is less likely to occur, as the lipidic vesicle is expected to fuse in the sebum content of the HF, it cannot be discarded entirely.

For the LP PC Chol, the targeting value increases from 0.15 in 12 h to 0.27 after 24 h, in which a kind of equilibrium is reached regarding the DUT amount in all the analyzed skin layers. The same behavior is not observed for the LP PC formulation, which remains with almost the same targeting factor after 12 and 24 h (0.14 and 0.13, respectively). This is probably because of the slower drug release of DUT from these formulations, which may require a longer time to reach such an equilibrium.

DUT control did not target HFs in 12 h (follicular targeting = 0.20), but it also reached equilibrium in 24 h (follicular targeting = 0.35), not significantly higher than LP Chol and TF (*p* > 0.005). Notwithstanding, no DUT was detected in the receptor compartment (LOD = 0.14 µg/mL) for any tested drug delivery system and control.

## 4. Conclusions

Here, we obtained DUT-loaded liposomes with different bilayer structures, which impacted the drug release profile, DUT accumulation in the skin following a permeation experiment, and drug targeting to the HFs. The addition of cholesterol to the bilayer structure, expected to improve vesicle stiffness, resulted in a faster drug release, as the cholesterol itself may have competed with the DUT for the lipidic interaction sites. Such behavior also affected cutaneous penetration, promoting DUT penetration into the skin, not targeting the HFs. The addition of an edge activator to the liposome bilayer, forming a TF, which was expected to have a more flexible structure, had the opposite effect, promoting better control over DUT release and a higher targeting at the first hours. However, with time, DUT seemed to diffuse from the HF to the remaining skin, reaching an equilibrium between all the skin layers analyzed. Hence, TFs are a more suitable option for DUT topical delivery, but further formulation optimizations are needed. Further clinical studies should address the safety of the prolonged use of such formulations.

## Figures and Tables

**Table 1:** Liposome composition.

Liposomes	DUT 28 mM	PC 200 mM	Cholesterol 10 mM	Tween^®^80 200 mM
**LP PC**	0.25 mL	2 mL	-	-
**LP Chol**	0.25 mL	2 mL	0.25 mL	-
**TF**	0.25 mL	2 mL	-	0.225 mL

All formulations had a final DUT concentration of approximately 0.30 mg/mL.

**Table 2:** Liposome characteristics after filtration.

Liposomes	Size (nm)	PdI	Zeta Potential (mV)	EE (%)	DUT Concentration (mg/mL)
**LP PC**	347 ± 12.5	0.28 ± 0.04	−0.61 ± 0.25	73.5 ± 12.9	0.29 ± 0.03
**LP Chol**	285.7 ± 4.67	0.22 ± 0.02	1.63 ± 0.13	70.2 ± 10.5	0.28 ± 0.05
**TF**	212.3 ± 12.2	0.22 ± 0.02	1.27 ± 0.03	88.1 ± 1.0	0.35 ± 0.09

N = 3.

**Table 3:** Effect of filtration on liposome mean size (nm) and PdI.

	LP PC Unfiltered	LP PC Filtered	LP Chol Unfiltered	LP Chol Filtered	TF Unfiltered	TF Filtered
**Size (nm)**	863.4 ± 86.7	347 ± 12.5	2382.7 ± 271.0	285.7 ± 4.67	272.1 ± 28.3	212.3 ± 12.2
**PdI**	0.52 ± 0.10	0.28 ± 0.04	0.81 ± 0.16	0.22 ± 0.02	0.18 ± 0.03	0.22 ± 0.02

## Data Availability

Data can be provided by the authors upon reasonable request.
